# Receptor-binding domain-specific human neutralizing monoclonal antibodies against SARS-CoV and SARS-CoV-2

**DOI:** 10.1038/s41392-020-00318-0

**Published:** 2020-09-22

**Authors:** Fei Yu, Rong Xiang, Xiaoqian Deng, Lili Wang, Zhengsen Yu, Shijun Tian, Ruiying Liang, Yanbai Li, Tianlei Ying, Shibo Jiang

**Affiliations:** 1 College of Life Sciences, Hebei Agricultural University, Baoding, China; 2 Research Center of Chinese Jujube, Hebei Agricultural University, Baoding, China; 3 Key Laboratory of Medical Molecular Virology (MOE/NHC/CAMS), School of Basic Medical Sciences, Fudan University, Shanghai, China

**Keywords:** Drug discovery, Microbiology, Drug discovery, Microbiology, Drug discovery

## Abstract

The outbreaks of severe acute respiratory syndrome (SARS) and Coronavirus Disease 2019 (COVID-19) caused by SARS-CoV and SARS-CoV-2, respectively, have posed severe threats to global public health and the economy. Treatment and prevention of these viral diseases call for the research and development of human neutralizing monoclonal antibodies (NMAbs). Scientists have screened neutralizing antibodies using the virus receptor-binding domain (RBD) as an antigen, indicating that RBD contains multiple conformational neutralizing epitopes, which are the main structural domains for inducing neutralizing antibodies and T-cell immune responses. This review summarizes the structure and function of RBD and RBD-specific NMAbs against SARS-CoV and SARS-CoV-2 currently under development.

## Introduction

Coronaviruses represent a large family of viruses common in human and many different species of animals, including camels, cattle, cats, and bats. However, only a few of these viruses infect people and cause disease. The 2019 novel coronavirus (SARS-CoV-2), which causes Coronavirus disease 2019 (COVID-19), is the seventh known coronavirus to infect humans. The other 6 coronaviruses (HCoVs) are HCoV-229E, HCoV-OC43, HCoV-NL63, HCoV-HKU1, SARS-associated coronavirus (SARS-CoV) and Middle East Respiratory Syndrome (MERS-CoV). SARS-CoV and MERS-CoV cause very severe symptoms, while the other four viruses usually cause only mild to moderate upper respiratory disease in humans.

**Fig. 1:**
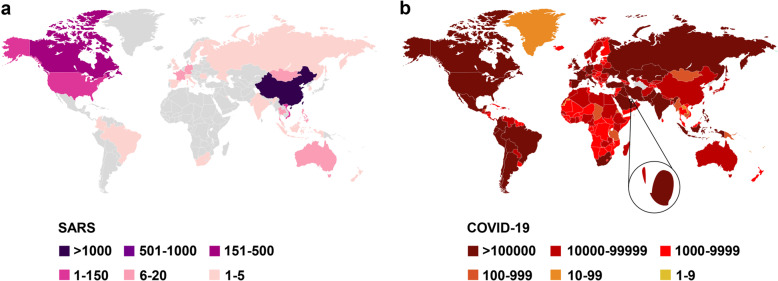
Statistical charts of cumulative cases. Statistical charts of cumulative number of SARS cases (**a**) and COVID-19 cases (**b**)

Severe acute respiratory syndrome (SARS) is a viral respiratory illness caused by SARS-CoV, the first worldwide pandemic caused by a coronavirus. Before it was contained, SARS had affected 32 countries in North America, South America, Europe, and Asia. As of December 31, 2003, the total number of SARS cases worldwide had reached 8096, with 774 deaths and a fatality rate of about 9.6% (https://www.who.int/csr/sars/country/table2004_04_21/en/). COVID-19 refers to the respiratory symptoms and pneumonia caused by SARS-CoV-2 infection. By Sep 18, 2020, according to WHO, SARS-CoV-2 had resulted in 30,369,778 confirmed cases and 948,795 deaths worldwide (https://www.who.int/emergencies/diseases/novel-coronavirus-2019). The total number of COVID-19 cases may, however, be higher owing to the inherent difficulties in identifying and counting mild and asymptomatic cases. In addition, the capacity to detect COVID-19 remains inadequate. Some characteristics of SARS-CoV and SARS-CoV-2 are compared in this review (Table 1). We also provide statistics on the distribution of these two epidemics in countries around the world (Fig. 1).

**Table 1:** Characteristics of SARS-CoV and SARS-CoV-2

Characteristics	SARS-CoV	SARS-CoV-2
Species	β Coronavirus, beta-CoV, lineage B	β Coronavirus, beta-CoV, lineage B
Origin	Bat	Bat
Intermediate hosts	Not known	*Manis pentadactyla*^68^
Genetic material	Positive-stranded RNA	Positive-stranded RNA
Receptor	Angiotensin-converting enzyme 2 (ACE2)	Angiotensin-converting enzyme 2 (ACE2)
Route of transmission	Respiratory droplets, close contact, fecal-oral route (?)	Respiratory droplets, close contact, fecal-oral route (?)
Incubation period	5 (2–10) days (https://www.who.int/csr/sars/postoutbreak/en/)	5.2 (1.8–12.4) days^69^
Clinical symptoms	Fever (99%), Documented elevated temperature (85%), Nonproductive cough (69%), Myalgia (49%), Dyspnea (42%)^70^	Fever (83%), Cough (82%), Shortness of breath (31%), Muscle ache (11%), Confusion (9%), Headache (8%), Sore throat (5%), Rhinorrhoea (4%), Chest pain (2%), Diarrhea (2%), Nausea and vomiting (1%)^71^
Fatality rate	9.6%	~3.1%^a^
Epidemic area	Asia, Europe, North, and South America	Globally
Epidemic time	~8 months	Unknown
High-risk for severe illness	Young and middle-aged	Elderly or frail with underlying disease
Possibility of recurrence	Very unlikely	Very likely

^a^Calculated based on the total number of the confirmed cases and deaths as of Sep 19, 2020

Treating with small-molecule compounds has shown good clinical effect, but we still need to be mindful of side effects which play an important role in the selection of therapeutic methods and reagents. Currently, no specific drugs can completely cure the disease caused by SARS-CoV-2. Therefore, it is urgent to develop safe and effective therapeutic reagents. Fortunately, human antibodies can accurately and efficiently identify antigens, and they have few side effects in humans. Antibody drugs are widely used in many fields, including cancer and infectious diseases. In addition, antibodies against SARS-CoV and MERS-CoV have been extensively developed.^1^

## Structure and function of the RBD in SARS-CoV and SARS-CoV-2 spike protein

**Fig. 2:**
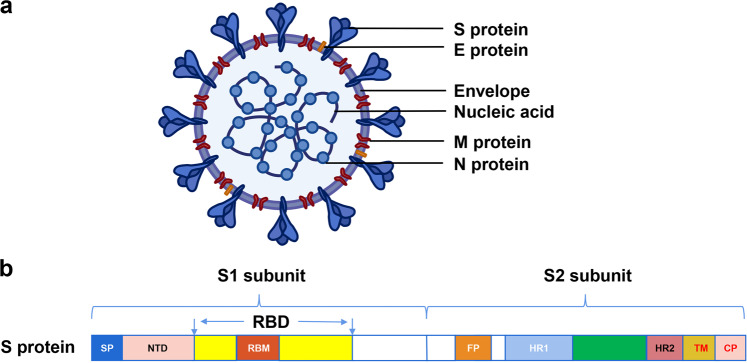
Schematic diagram of coronavirus particle and S protein gene partitioning. (**a**) Major structural proteins of the coronavirus particle include Spike glycoprotein (S protein); Membrane (M) protein; Nucleocapsid (N) protein; and Envelope (E) protein. (**b**) The S protein is mainly divided into S1 and S2 subunits and subdivided again into signal peptide (SP); N-terminal domain (NTD); receptor-binding domain (RBD), which contains receptor binding motif (RBM); fusion peptide (FP); heptad repeat region 1 (HR1); heptad repeat region 2 (HR2); transmembrane region (TM); and cytoplasmic tail (CP) according to different functions

**Fig. 3:**
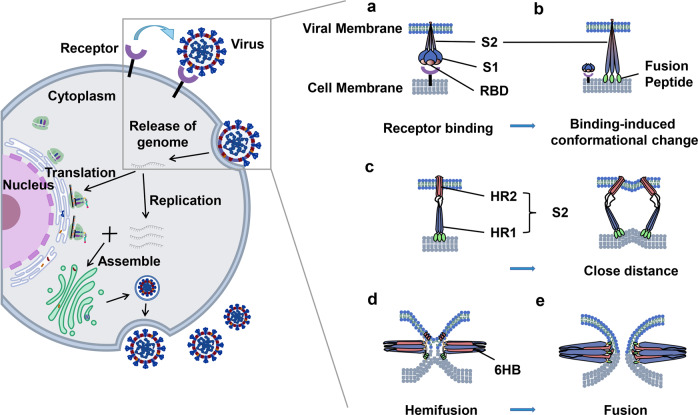
Schematic diagram of coronavirus life cycle. The virion enters the cell through recognizing specific receptor, uses the host cell resources to carry on genome replication and synthesis of major structural proteins, and then finally assembles into the mature virion which is then released out of cell. The invasion process mainly includes (**a**) coronavirus S protein recognition and binding to cell receptors, along with virus attachment to the cell; (**b**) induction of conformational changes in the S protein upon binding, in which the fusion protein is exposed and inserted into the host cell membrane; (**c**) HR1 and HR2 of the S2 subunit gradually approaching each other, narrowing the distance between viral envelope and host cell membrane; (**d**, **e**) HR1 and HR2 forming a six helical bundle (6-HB), which causes the virus envelope and host cell membrane to fuse from the hemifusion state to complete fusion, thus releasing the virus gene into the host. Viral genes replicate and translate within cells to produce genomic RNA and viral proteins, which are assembled with the proteins to form viral particles

**Fig. 4:**
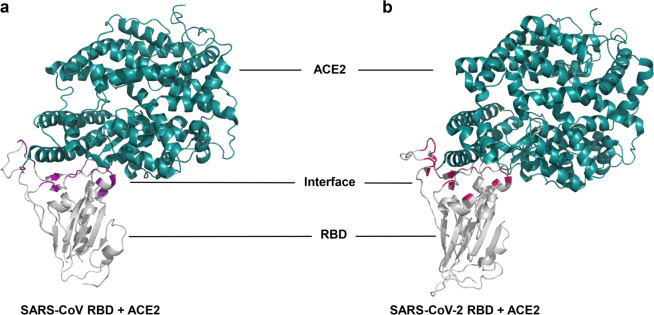
Crystal structure of RBD binding to ACE2. (**a**) RBD (gray) of SARS-CoV specifically binds the receptor ACE2 (dark green) (PDB: 2AJF), and the interface is marked purple; (**b**) RBD (gray) of SARS-CoV-2 specifically binds the receptor ACE2 (PDB: 6LZG), and the interface is marked hot pink

Coronaviruses are enveloped spherical particles, the spike glycoproteins (S protein) of which form a crown-like surface (Fig. 2a). The S protein consists of two subunits: S1 and S2 (Fig. 2b). The fragment located in the middle of the S1 subunit (amino acids (aa) 318–510) is the minimum receptor-binding domain (RBD) in SARS-CoV, which binds to the host cell receptor angiotensin-converting enzyme 2 (ACE2).^2–4^ The binding of RBD and ACE2 triggers the conformational change of the S2 subunit and virus particle invasion^5^ (Fig. 3). The crystal structure of the RBD bound to ACE2 peptidase indicates that RBD presents a flat concave surface at the N-terminus of the receptor peptidase on which aa 445–460 anchor the entire receptor-binding loop of the RBD core.^6^ This loop (aa 424–494) makes complete contact with the ACE2 receptor and is called the receptor binding motif (RBM)^6^ (Fig. 2b). Scientists have already developed safe and effective vaccine candidates against SARS-CoV based on the RBD and screened neutralizing antibodies using it as an antigen. These studies indicate that the RBD contains multiple conformational neutralizing epitopes, which are the main structural domains for inducing neutralizing antibodies and T-cell immune responses, making the RBD an important target for the development of vaccines and immunotherapeutics.^7–9^ Since the emergence of SARS-CoV, researchers have screened, or designed, many NMAbs targeting the RBD. Following the outbreak of COVID-19, the sequence of SARS-CoV-2 was soon identified, and it was found that the structure and function of its RBD are very similar to those of SARS-CoV. Moreover, the cell receptor for both viruses is ACE2,^10,11^ and the crystal structure of both virus RBDs interacting with ACE2 is shown in Fig. 4. Therefore, some NMAbs targeting SARS-CoV RBD may also have potential neutralizing activity against SARS-CoV-2. This means that researchers can use the SARS-CoV-2 RBD to screen for NMAbs and design vaccines against SARS-CoV-2. Most recently, Yang et al have reported that a SARS-CoV-2 RBD-based recombinant COVID-19 vaccine could induce potent neutralizing antibody responses in the immunized mice, rabbits, and NHPs, and provided protection in non-human primates (NHPs) against SARS-CoV-2 challenge,^12^ highlighting the importance of the RBD in development of neutralizing antibodies and vaccines.

### RBD-specific human NMAbs against SARS-CoV

**Fig. 5:**
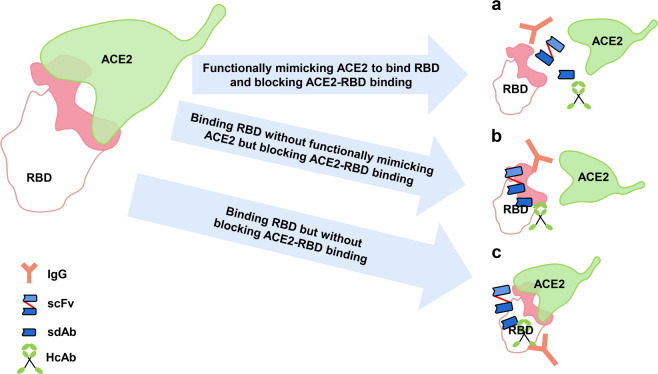
Pattern of NMAbs action. The major types of neutralizing monoclonal antibodies (NMAbs) include immunoglobulin G (IgG), single-stranded variable region fragments (scFvs), heavy-chain antibody (HcAb) and single-domain antibodies (sdAbs). NMAbs exert antiviral activity by (**a**) NMAbs functionally mimicking ACE2 to bind RBD and blocking ACE2-RBD binding; (**b**) NMAbs binding RBD without functionally mimicking ACE2 but blocking ACE2-RBD binding; (**c**) NMAbs binding RBD but without blocking ACE2-RBD binding (interface is marked pink)

NMAbs targeting SARS-CoV RBD generally prevent the attachment of the virus to the host cell by interfering with the binding of viral RBD to cellular ACE2 receptor (Fig. 5). These RBD-targeted NMAbs can be roughly divided into the following three categories based on their different binding sites on RBD. First, NMAbs that bind to the ACE2-binding site on RBD, including the receptor-binding motif (RBM), can functionally mimic ACE2 to bind RBD and block ACE2-RBD binding (Fig. 5a). Second, NMAbs that bind to the epitope outside or partly overlap ACE2-binding site on RBD can indirectly block ACE2-RBD binding without functionally mimicking ACE2 to bind RBD (Fig. 5b). Third, NMAbs that bind to the epitope outside ACE2-binding site on RBD and could not block ACE2-RBD binding (Fig. 5c).

### NMAbs functionally mimicking ACE2 to bind RBD and blocking ACE2-RBD binding

**Fig. 6:**
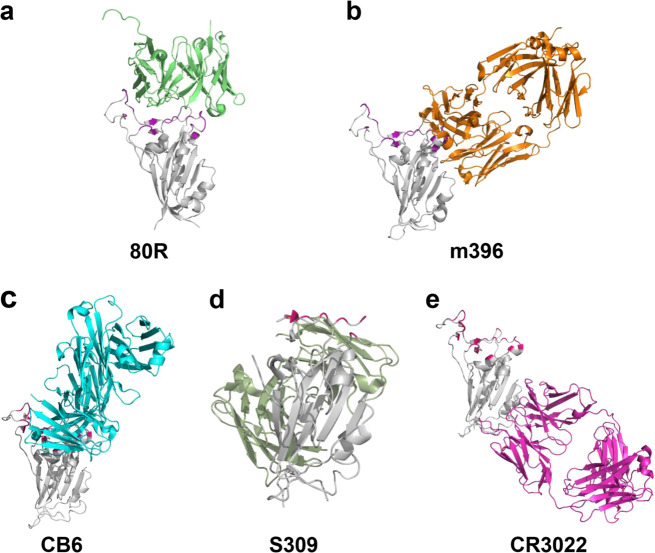
Crystal structure of NMAb binding to RBD. (**a**) 80 R (light green) imitates ACE2 binding to the interface (purple) on RBD of SARS-CoV (PDB: 7BZ5); (**b**) m396 (orange) binds to RBD of SARS-CoV without functionally mimicking ACE2, preventing RBD from binding to ACE2 (PDB: 2DD8). (**c**) CB6 (sky blue) functionally mimics ACE2 binding to the interface (hot pink) on RBD of SARS-CoV-2 (PDB: 7C01); (**d**) S309 (grass green) binds to RBD of SARS-CoV-2 without blocking the binding of RBD to ACE2 (PDB: 6WPS). (**e**) The SARS-CoV-specific NMAb CR3022 (purple) binds to RBD of SARS-CoV-2 without blocking the binding of RBD of SARS-CoV-2 to ACE2 (PDB: 6W41)

In the development of antibodies against SARS-CoV, eight (6A, 8C, 12E, 26H, 27D, 80R, 91M, and 92N) recombinant human single chain variable fragments (scFvs) targeting the S1 of SARS-CoV S glycoprotein were identified from two nonimmune phage libraries of human antibodies.^13^ One of them, 80R, effectively neutralized SARS-CoV (Urbani strain) in vitro. 80R scFv competes with soluble ACE2 to bind the RBD in the S1 domain (Fig. 6a) with high affinity (equilibrium dissociation constant Kd = 32.3 nM). At a concentration of 7.43 nM, 80R scFv can neutralize >50% of the testing wells from SARS-CoV infection. In most cases, the bivalent full-length immunoglobulin is more effective than its corresponding scFv because of avidity, effector functions, and prolonged serum half-life. The same neutralizing activity was achieved by 80R IgG1 at a concentration as low as 0.37 nM. Moreover, 80R IgG1 inhibits syncytial formation between cells expressing S protein and cells expressing ACE2.^13^ In a further study, 80R IgG1 could efficiently protect mice from SARS-CoV infection, noting that the core of its target is aa 324 to 503 in the S protein of SARS-CoV.^14^ This is also the first effective antibody against SARS-CoV screened from an antibody library.

CR3014 was also identified from semisynthetic antibody phage display libraries. The results showed that CR3014 targets residues 318 to 510, a region previously identified as the SARS-CoV RBD. Furthermore, complete protection against 100 TCID_50_ (50% tissue culture infectious dose) of SARS-CoV infectivity was achieved at 42 nM of CR3014 IgG.^15^ At the same time, researchers aligned residues 318–510 of the S protein from 114 human SARS-CoV isolates published in GenBank.^15^ The results showed that eight sequences of the S protein were different from those used in the beginning of this article. These sequences were expressed in the form of recombinant S318–510 fragments. Among them, CR3014 had no neutralizing effect on the N479 mutant sequence, indicating that this residue is very important for the binding of CR3014.^15^ In a follow-up study, CR3014 IgG suppressed replication of SARS-CoV in the lungs of infected ferrets, attenuated SARS-CoV-induced visible lung lesions, and eliminated virus reproduction in the pharyngeal region at 10 mg/kg.^16^

To study the inactivated virus as a potential vaccine, inactivated SARS-CoV particles were used as the immunogen to induce humoral and/or cell-mediated immune response in mice, and antibodies were detected in the hybridoma supernatant. Two NMAbs (1A5, 2C5) with strong neutralizing effects on SARS-CoV were screened out. The results of virus neutralization experiments showed that 1A5 (32 µg/µl) and 2C5 (14 µg/µl) were diluted 45,000 and 11,000 times to achieve IC_50_ values of ~0.71 µg/ml and 1.27 µg/ml, respectively. When mapping epitopes of NMAbs on specific domains of S protein, both NMAbs were found to target residues 310–535 in S protein of SARS-CoV.^17^

By imitating the human immune response to SARS-CoV, S230.15 was identified by B cell-based Epstein-Barr virus (EBV) transformation and used the RBD of S protein as antigen. S230.15 competes with SARS-CoV receptor ACE2 to bind to the RBD of the S protein. S230.15 potently inhibited viral entry into cells and cell fusion mediated by the S protein of SARS-CoV in vitro. Furthermore, the antiviral activity of S230.15 was tested in mice infected with recombinant SARS-CoV. Mice infected with Urbani and GD03 were treated with 200 µg of S230.15, and the mice were completely protected.^18^

### NMAbs binding RBD without functionally mimicking ACE2 but blocking ACE2-RBD binding

As mentioned before, m396 was identified from healthy human antibody libraries of volunteers by using the RBD of SARS-CoV S protein as antigen.^19^ Further study showed that m396 by targeting the RBD (Fig. 6b), m396 could neutralize GD03 and Tor2 viruses with IC_50_ of 0.1 µg/ml and 0.01 µg/ml, respectively, and neutralize pseudoviruses from the Urbani isolate. Mice infected with Urbani and GD03 were treated with 200 µg m396, and the mice were completely protected. Meanwhile, m396 showed significant, but incomplete, protection against SZ16 infection in mice. By analyzing the key residues of m396 on RBD of SARS-CoV, Zhu et al found that the epitope of m396 overlapped the binding site of ACE2, including residues Y484, T486, T487, G488, Y491.^18^ Therefore, m396 neutralizes viruses by competing with ACE2 to bind to RBD.

In addition to the humanization of mouse antibodies, transgenic technology can replace the mouse immunoglobulin gene with human immunoglobulin gene, and animal immunization with antigen can obtain full human antibodies. Following this practice, a fully human antibody, 201, was developed, that targets residues 490–510 of the SARS-CoV S protein. Affinity measurements showed an affinity Kd of 34 nM between monoclonal antibodies (mAbs) and soluble S_590_ (residues 1–590 of the SARS-CoV S protein fragment consisting of residues 1–590). The NMAb 201 specifically blocked binding of S_590_ to Vero E6 cells in a flow cytometry –based assay and. 201 provided a 50% maximal protective effect at concentrations of ~1 nM in the neutralization assays against SARS-CoV in vitro. The NMAb 201 completely protected mice against SARS-CoV infection at a concentration of 40 mg/kg. In doses as low as 1.6 mg/kg, mice could be effectively protected, even though replication of virus was not completely inhibited in the lungs.^20^

NMAbs were produced from mice immunized with inactivated SARS-CoV (Tor-3 strain, isolated from a patient infected in the Toronto SARS outbreak^21^) using the hybridoma technique. The most potent mAbs, including F26G9, F26G10, F26G18, and F26G19, were screened out in ELISA assays.^22^ These mAbs were then humanized and tested for their affinity and neutralization activity to SARS-CoV. Results showed that chimeric F26G18 bound full-length S protein (rFS) with a Kd of 1.78 ± 0.63 nM and that chimeric F26G18 had a neutralization titer of 2.47 nM which matched that of parental F26G18 with a neutralization titer of 2.07 nM against heat inactivated Tor2.^23^ Also, ELISA assays were performed to determine S-protein and RBD specificity for the different mAbs. Of these mAbs, chimeric F26G18 bound to the SARS-CoV RBD, and the reaction was independent of glycosylation. Furthermore, F26G18 and its chimera mapped to a discrete sequential epitope (_460_FSPDGKPCTPPALNCYW_476_), which overlaps the ACE2 binding site, in the RBD of SARS-CoV S protein,^23^ suggesting that F26G18 neutralizes SARS-CoV infection by blocking RBD-ACE2 the binding.

Using hybridoma technology, 26 hybridomas were screened out from mice immunized with radiation-inactivated SARS-CoV. Nine of the most representative clones were selected for further identification. The results showed that five (154C, 240C, 341C, 534C, 560C) of these clones targeted the S protein and that NMAb 341C recognized residues 490–510 in the SARS-CoV S protein. Furthermore, the results of neutralization experiments showed that 341C had the strongest neutralizing activity, completely inhibiting the lysis of virus-infected Vero E6 cells at a dilution of 1: 1280.^24^

In a recent study, five variable heavy homodimers (VHH) (VHH-1, VHH-6, VHH-35, VHH-44, and VHH-72) were screened out from a llama immunized with the S protein of SARS-CoV. To assess the antiviral activity of these mAbs, neutralization assays were performed in vitro using SARS-CoV Urbani S pseudotyped lentiviruses. The results showed that VHH-72 neutralized the pseudovirus with an IC_50_ value of 0.14 µg/ml. Epitope mapping showed that VHH-72 targets the RBD of SARS-CoV S protein, and the crystal structure shows that VHH-72 blocks the binding of ACE2 and RBD through steric hindrance.^25^

### NMAbs binding RBD but without blocking ACE2-RBD binding

The genome of SARS-CoV continues to mutate during replication, and many virus subtypes already exist. Therefore, the targets of antibodies need to extensively cover different SARS-CoV isolates and control potential neutralization escape mutations for effective human immune prophylaxis. Combinations of neutralizing and noncompetitive mAbs may have these properties.^26^

The previously mentioned CR3014 IgG does not work in all SARS-CoV strains. In order to cover the remaining virus strains, CR3022 was identified from an immune scFv phage display library constructed from the lymphocytes of a convalescent SARS patient from Singapore. According to the study, all CR3014 IgG escaped virus showed a single point mutation at amino acid position 462 (proline to leucine, P462L) in the S protein. CR3022 IgG does not compete with CR3014 in combination with recombinant S1 fragments. CR3022 IgG completely neutralized the SARS-CoV strain HKU-39849 and the CR3014 IgG escape variants at a concentration of 23.5 µg/ml. The synergistic effect of CR3014 IgG and CR3022 IgG may also reduce the total antibody dose of passive immunity against SARS-CoV infection.^26^

Transgenic H2L2 mice, which encode chimeric immunoglobulin, including variable regions of human heavy and light chains and constant regions of rats, were immunized with the S protein of SARS-CoV. Four hybridomas that activated on the S protein of SARS-CoV were identified. Among them, the chimeric antibody 47D11 showed neutralization activity to SARS-CoV pseudovirus. 47D11 was then reconstructed and expressed in the form of human IgG1 and further identified. The results showed that the humanized antibody 47D11 could bind the SARS-CoV S protein expressed on the cell surface and inhibit the infection of SARS-CoV pseudovirus with an IC_50_ value of 0.061 μg/ml. Infection of Vero E6 cells with SARS-CoV was neutralized with an IC_50_ value of 0.19 μg/ml. 47D11 was shown to target the RBD of SARS-CoV in an ELISA assay.^27^

NMAb S309 was identified from the peripheral blood of SARS- infected patients. Neutralization assays showed that antibody S309 could effectively neutralize pseudoviruses carrying the S proteins of SARS-CoV isolates from the early, middle and late phases of the 2002–2003 epidemic with IC_50_ values between 120 and 180 ng/ml. S309 targets the front end of the RBD of the SARS-CoV S protein.^28^

### RBD-specific human NMAbs against SARS-CoV-2

Since SARS-CoV-2 and SARS-CoV share the receptor ACE2 and their RBDs have similar sequences, the NMAbs against SARS-CoV-2 can be classified in the same way as those against SARS-CoV (Fig. 4).

### NMAbs functionally mimicking ACE2 to bind RBD and blocking ACE2-RBD binding

Five (1E2, 2F2, 3F11, 4D8, and 5F8) single-domain antibodies (sdAbs) with antiviral activity to SARS-CoV-2 in vitro were identified from a fully synthetic, humanized phage display library with recombinant RBD of the SARS-CoV-2 S protein. In a pseudotyped virus neutralization assay, monomeric sdAb 3F11 showed neutralization activity with an IC_50_ of 0.0038 µg/ml. 3F11 also neutralized SARS-CoV-2 with an IC_50_ value of 0.4360 µg/ml in a live virus neutralization assay. The results of a competitive ligand-binding assay suggested that 3F11 completely blocked the binding between SARS-CoV-2 RBD and ACE2.^29^ To overcome the limitations of monovalent sdAbs,^30^ sdAbs was fused with human IgG1 Fc fragments. Results showed that the neutralization activity of recombinant 3F11 significantly increased by 10.8-fold in molar concentration with an IC_50_ value of 0.0020 µg/ml in a SARS-CoV-2 pseudotyped virus entry assay.^29^

Fourteen effective NMAbs against SARS-CoV-2 were identified from 60 convalescent patients using high-throughput single-cell RNA and VDJ sequencing of antigen-enriched B cells. Among them, BD-368-2 had the most potency, effectively neutralizing the pseudovirus of SARS-CoV-2 and live SARS-CoV-2 with an IC_50_ of 1.2 ng/ml and 15 ng/ml, respectively. In SARS-CoV-2-infected transgenic mice, BD-368-2 also showed strong therapeutic and prophylactic effects. In addition, the target verification results showed that the epitopes of BD-368-2 overlapped with the ACE2 binding site, and the crystallographic analysis indicates that BD23-Fab blocks the interaction between RBD and ACE2.^31^

In addition, ab1 was identified from eight large phage displayed Fab, scFv and VH libraries by panning against the RBD of the SARS-CoV-2 S protein. ab1 competes with hACE2 (human ACE2) for binding to RBD, indicating that ab1 neutralizes SARS-CoV-2 infection by blocking RBD-ACE2 binding. The ab1 IgG1 could completely neutralize SARS-CoV-2 in a concentration of <400 ng/ml. Moreover, it protected transgenic mice expressing hACE2 from a high-titer intranasal SARS-CoV-2 challenge. A relatively low number of somatic mutations occurred in the sequence of ab1, as determined by sequence alignment results. This indicates that either natural SARS-CoV-2 infection or RBD-based vaccines could stimulate the quick production of ab1-like antibodies. The ab1 IgG1 showed no safety deficiencies; therefore, it has therapeutic and prophylactic potential for SARS-CoV-2 infections.^32^

Recently, CB6 was identified from the PBMCs of a COVID-19 convalescent patient by using recombinant RBD of the SARS-CoV-2 S protein. CB6 binds RBD (Fig. 6c) and blocks the binding of soluble SARS-CoV-2-RBD with hACE2. Both pseudovirus and live virus neutralization assays were performed to investigate the neutralization activity of CB6 against SARS-CoV-2 in vitro. The results showed that CB6 could inhibit the pseudovirus of SARS-CoV-2 with ND_50_ (50% neutralization dose) values of 0.036, 0.023 and 0.041 μg/ml in Huh7, Calu-3, and HEK293T cells, respectively. CB6 could also effectively neutralize SARS-CoV-2 with an ND_50_ of 0.036 ± 0.007 μg/ml in infected Vero E6 cells. Furthermore, CB6 was tested in a rhesus macaque model infected with SARS-CoV-2 in both prophylactic and treatment settings and showed a considerable effect. Investigating the neutralization mechanism showed that CB6 could block the binding of hACE2 to RBD through steric hindrance and competition with interface residues.^33^

Similarly, two NMAbs, B38 and H4, were isolated from a convalescent COVID-19 patient in China and were recently reported to have antiviral activity against SARS-CoV-2. Similar to Cao et al.,^31^ the RBD of SARS-CoV-2 was used as bait to isolate specific single memory B cells from PBMC of the patient. In order to identify the antiviral activity of these isolated mAbs, a virus neutralization experiment against SARS-CoV-2 (BetaCoV/Shenzhen/SZTH-003/2020) was performed. The results showed that B38 had higher potency to inhibit viral infection with an IC_50_ value of 0.177 μg/ml. This was followed by H4, which inhibited viral infection with an IC_50_ value of 0.896 μg/ml. The crystal structure of the RBD-B38 complex shows that most of the residues on the B38 binding epitope overlap with the RBD-ACE2 binding interface, suggesting that B38 neutralizes SARS-CoV-2 infection by functionally mimicking ACE2 to bind RBD and block the RBD-ACE2 binding. B38 and H4 competed with ACE2 for RBD binding in the competition assay. In animal trials, lung viral load was reduced by 32.8% and 26% with a single dose of 25 mg/kg of B38 and H4, respectively, compared with the untreated group. Similarly, a competition assay was performed to identify whether B38 and H4 act on the same epitope on the RBD. No competition was observed between B38 and H4, suggesting that the two NMAbs acted on different RBD sites. Therefore, B38 and H4 can be used as an ideal mAb pair for virus targeting to avoid immune escape in future clinical applications.^34^

### NMAbs binding RBD without functionally mimicking ACE2 but blocking ACE2-RBD binding

Similarly, another NMAb, 7B11, was identified from previously screened antibodies. It is specific to SARS-CoV. ELISA assays were performed to detect the binding activity against the RBD of SARS-CoV-2, and the results showed that six mAbs (46C1, 13B6, 29H4, S29, 7B11, and 18F3) had cross-reactivity against SARS-CoV-2 RBD. 7B11 showed cross-neutralization activity against SARS-CoV-2 in a pseudovirus neutralization assay, neutralizing about 80% of pseudovirus at 10 μg/ml. According to this report, 7B11 could block the binding of SARS-CoV and SARS-CoV-2 RBDs to ACE2 owing to proximity between target and binding site.^35^

In order to rapidly obtain human NMAbs targeting SARS-CoV-2, 206 mAbs specific to the RBD of SARS-CoV-2 S protein were screened from the blood of eight SARS-CoV-2-infected individuals. Analysis of 13 of the 69 antibodies from patient two showed neutralization activity of P2C-1F11 and P2B-2F6 against the SARS-CoV-2 pseudovirus with IC_50_ values of 0.03 and 0.05 µg/ml, respectively. Consistent with experimental results of the pseudovirus neutralization, P2C-1F11 and P2B-2F6 neutralization of SARS-CoV-2 demonstrated the most potent neutralization activity with IC_50_ values of 0.03 and 0.41 μg/ml, respectively.^36^

A competitive biopanning strategy was developed to effectively screen RBD blocking antibodies from the phage display antibody library. rRBD-15 and rRBD-16, with the highest affinity for RBD screened from competitive biopanning, were expressed as a full-length IgG1. They can bind to the RBD with EC_50_ values at 3.8 nM and 5.3 nM, respectively in vitro. Only rRBD-15 inhibited the binding of RBD to ACE2 with an IC_50_ at 3.0 nM, while rRBD-16 had no blocking or neutralizing activity. In a pseudovirus neutralization assay, rRBD-15 showed potent neutralization activity against SARS-CoV-2 pseudovirus infection with an IC_50_ of 12.2 nM.^37^

VHH-72 has been shown to have cross-reactivity between SARS-CoV and SARS-CoV-2 S protein. Experimental data showed that the binding affinity of VHH-72 to the SARS-CoV-2 RBD was ~39 nM, which was weaker than that of the SARS-CoV RBD. According to the report, this was related to the rapid dissociation of VHH-72. Among the amino acid residues of RBD binding to VHH-72, the only mutation found was Arg426, which is Asn439 in the SARS-CoV-2 RBD. However, the binding of VHH-72 to RBD of SARS-CoV-2 could not be detected in ELISA assays, and VHH-72 could not neutralize SARS-CoV-2 pseudovirus. In order to overcome the rapid dissociation of VHH-72, scientists modified VHH-72, including a tail-to-head fusion of two VHH-72 molecules connected by a (GGGGS)3 linker (VHH-72-VHH-72) and a genetic fusion of VHH-72 to the Fc domain of human IgG1 (VHH-72-Fc). Both variants exhibited binding ability to the S proteins of SARS-CoV and SARS-CoV-2, and VHH-72-Fc neutralized SARS-CoV-2 pseudovirus with an IC_50_ of ~0.2 µg/ml.^25^

To investigate the implications of a SARS-CoV-2 RBD-hACE2 interaction, Chen et al.^38^ first identified the inhibitory activity of plasma from recovered patients and then sorted out the memory B cells of positive plasma samples. Using reverse transcription polymerase chain reaction, they next cloned the VH and VL of IgG which were expressed in vitro again, finally obtaining three mAbs (311mab–31B5, 311mab–32D4 and 311mab–31B9). Consistent with plasma test results, 311mab-31B5 and 311mab-32D4 could efficiently block the SARS-CoV-2 RBD-hACE2 interaction with an IC_50_ of 0.0332 and 0.0450 μg/ml, respectively, while 311mab–31B9 had no inhibitory activity against this interaction. Both 311mab-31B5 and 311mab-32D4 showed neutralizing activity against SARS-CoV-2 pseudovirus with an IC_50_ of 0.0338 and 0.0698 μg/ml, respectively, while 311mab-31B9 had no neutralization activity in this assay.^38^

In another study,^39^ common mAbs were screened out from convalescent patients and specific mAbs were obtained using high-throughput screening with specific antigen. A neutralization assay for the pseudovirus of SARS-CoV-2 and a protection assay in Syrian hamsters were performed to examine the antiviral activity of mAbs. Among the identified mAbs, CC12.1 effectively neutralized the pseudovirus of SARS-CoV-2 with an IC_50_ value of 0.019 µg/ml and live SARS-CoV-2 with an IC_50_ value of 0.022 µg/ml. CC12.1 could completely protect Syrian hamsters against the Washington strain USA-WA1/2020 (BEI Resources NR-52281) in a plasma concentration of 22 µg/ml. Furthermore, CC12.1 was found to target the RBD of SARS-CoV-2 and block ACE2 binding in a mapping assay. In addition, CC6.29 and CC6.30, which target the RBD of SARS-CoV-2, have the highest potency against SARS-CoV-2 pseudovirus with IC_50_ values of 2 ng/ml and 1 ng/ml, respectively.^39^

Similarly, COVA1-18 and COVA2-15 were identified from convalescent patients by using SARS-CoV-2 S protein as antigen. Both NMAbs targeted the RBD of SARS-CoV-2 S protein.^40^ These mAbs showed neutralization activity against the pseudovirus of SARS-CoV-2 with an IC_50_ value of 8 ng/ml and potently inhibited live SARS-CoV-2 infection with IC_50_ values of 7 and 9 ng/ml, respectively.

To obtain a broad-spectrum vaccine that effectively targets known and potential human coronaviruses, eight NMAbs with SARS-CoV-2 cross-neutralizing activity were identified by using a memory B cell repertoire from a convalescent SARS donor. It is worth noting that these NMAbs can effectively cross-neutralize SARS-CoV, SARS-CoV-2 and the bat SARS-like virus WIV1. ADI-55689 binds to the edge of the ACE2 binding site, close to the more conservative core region of the RBD structure, while ADI-56046 binds to the flexible tip of RBD. The IgG format of these NMAbs showed neutralizing activity against SARS-CoV-2 live virus at 100 nM, and similar results were shown in a VSV-based pseudovirus neutralizing assay. In a MLV-based pseudovirus neutralizing assay, ADI-55689 and ADI-56046 showed neutralizing activity with IC_50_ values of 0.05–1.4 μg/ml and 0.004–0.06 μg/ml against SARS-CoV-2 and SARS-CoV respectively. Similar IC_50_ values were observed in SARS-CoV-2 and SARS-CoV live virus neutralization assays.^41^

Using transgenic mice and PBMC of SARS-CoV-2-infected patients, a large number of single B cells that produced antibodies which specifically bind to SARS-CoV-2-RBD were screened out. The antibody genes of these B cells were then sequenced, and then expressed as complete antibodies. Finally, about 40 antibodies with unique sequences and neutralizing activity against SARS-CoV-2 pseudoviruses were obtained. Among of them, nine (REGN10989, REGN10987, REGN10933, REGN10934, REGN10977, REGN10964, REGN10954, REGN10984, REGN10986) of the most potent antibodies were identified in subsequent assays. Their IC_50_ values range from 7–99 pM. These antibodies can effectively block the binding of ACE2 to the RBD. In addition, four (REGN10989, REGN10987, REGN10933, REGN10934) of them can effectively neutralize the virus with IC_50_ values of 7.38, 42.1, 37.4, 28.3 pM, respectively. REGN10933 and REGN10987 can simultaneously bind to distinct regions of the RBD through crystal structure analysis. Therefore, these two NMAbs can be used as cocktails to exert antiviral activity against SARS-CoV-2. Human trials (clinicaltrials.gov NCT04426695 and NCT04425629) of an antibody cocktail are being tested now.^42^ In addition, these antibodies were combined to explore their effect on escape mutations. The results showed that this noncompetitive antibody cocktail therapy can effectively avoid viral escape mutations.^43^

A single-domain antibody H11 that specifically binds to SARS-CoV-2 RBD was identified by screening a naive llama phage display antibody library. Through random mutation, two mutants H11-H4 and H11-D4 H11 that have higher affinity for SARS-CoV-2 RBD were obtained. Bivalent Fc-nanobody fusion, H11-H4-Fc, and H11-D4-Fc, compete with ACE2 for RBD binding and H11-H4 and H11-D4 recognize the same epitope, which partly overlaps to the ACE2 binding region in the SARS-CoV-2 RBD. H11-H4-Fc and H11-D4-Fc can block the binding of RBD to MDCK-ACE2 with IC_50_ of 61 nM for H11-H4-Fc, and 161 nM for H11-D4-Fc, respectively. The results from the plaque reduction neutralization assay showed that H11-H4-Fc and H11-D4-Fc could neutralize live SARS-CoV-2 infection with the ND_50_ of 6 nM and 18 nM, respectively. Combining CR3022 with H11-H4-Fc or H11-D4-Fc exhibited synergistic effect on their neutralization effect.^44^

As the pandemic continues, more and more NMAbs have been screened out. A humanized NMAb H014 was identified from a phage display antibody library generated from RNAs extracted from peripheral lymphocytes of mice immunized with SARS-CoV RBD, which could cross-neutralize SARS-CoV and SARS-CoV-2. Both IgG and Fab forms of H014 could effectively bind to the RBD of SARS-CoV and SARS-CoV-2 at sub-nM levels. H014 IgG could effectively neutralize SARS-CoV-2 and SARS-CoV pseudovirus infection with IC_50_ of 3 and 1 nM respectively, and can neutralize the authentic SARS-CoV-2 virus with an IC_50_ of 38 nM. H014 IgG could effectively protect hACE2-Tg mice from the challenge with SARS-CoV-2. H014 IgG bound to the epitope outside RBM on RBD of SARS-CoV-2, thus, preventing RBD-ACE2 binding through steric hindrance.^45^

From the lymphocytes of SARS-CoV-2 convalescent patients in Wuhan, some NMAbs were identified from the screening with SARS-CoV-2 S protein.^46^ The IC_50_ of these NMAbs against SARS-CoV-2 strain WA1/2020 in a range of 15–4000 ng/ml as measured with focus reduction neutralization test. Among of them, COV2-2196 and COV2-2130 could also neutralize SARS-CoV-2 pseudovirus infection. They could completely block the binding between SARS-CoV-2 RBD and hACE2 in a competition assay, but bound to different epitopes on RBD. Therefore, COV2-2196 and COV2-2130 could simultaneously bind to S protein and exhibited synergistic neutralization effect on live SARS-CoV-2 infection. The use of COV2-2196 and COV2-2130 alone or in combination could protect BALB/c mice from challenge with a mouse-adapted SARS-CoV-2. Use of COV2-2196 or COV2-2381 alone could also protect NHPs from SARS-CoV-2 infection.^47^

Ho and colleagues isolated 61 NMAbs from PBMCs of five severe COVID-19 patients. Nine of them have IC_50_ in a range 0.7 ~ 9 ng/ml against live SARS-CoV-2 infection. Among of them, 2–15, 2–7, 1–57, and 1–20 targeted RBD, and 2–17, 5–24, and 4–8 targeted NTD, while 2–43 and 2–51 targeted a quaternary epitope on the top of RBD on the S protein trimer. The most potent NMAb 2–15 could neutralize pseudotyped and live SARS-CoV-2 infection with IC_50_ of 5 ng/ml and 0.7 ng/ml, respectively, by binding RBD, competitively with ACE2. The NMAb 2–15 could effectively protect a golden Syrian hamster model against SARS-CoV-2 infection.^48^

### NMAbs binding RBD but without blocking ACE2-RBD binding

As described in Wang et al.,^27^ transgenic mice were immunized with S protein of SARS-CoV, and NMAbs that have cross-reactivity against SARS-CoV-2 were identified. Among them, the chimeric antibody 47D11 was reconstructed, expressed in the form of human IgG1, and further characterized. The results showed that the humanized 47D11 could bind SARS-CoV-2 S protein expressed on the cell surface and inhibit the infection of SARS-CoV-2 pseudovirus with an IC_50_ value of 0.061 μg/ml. Infection of Vero E6 cells with SARS-CoV-2 was neutralized with an IC_50_ value of 0.57 μg/ml. 47D11 was shown to target the RBD of the SARS-CoV-2 S protein in an ELISA assay.^27^

18F3 was screened out from antibodies specific to SARS-CoV. ELISA assays were performed to detect the activity of binding to the RBD of SARS-CoV-2, and the results showed that six mAbs (46C1, 13B6, 29H4, S29, 7B11, and 18F3) had cross-reactivity against SARS-CoV-2 RBD. 18F3 showed cross-neutralization activity against SARS-CoV-2 in the pseudovirus neutralization assay, neutralizing about 80% of pseudovirus at 10 μg/ml. However, 18F3 could not block the binding of SARS-CoV and SARS-CoV-2 RBDs to ACE2 owing to the distance between target and binding site.^35^

As mentioned earlier, fully human antibodies have an advantage as drugs in human. In addition to camel origin and humanized sdAbs, fully human sdAbs have been reported.^49^ In this study, the S1 fragment was used as an antigen to screen sdAbs that specifically bound to the antigen from a fully human phage display library, followed by pseudovirus and live virus neutralization experiments, and a competition assay against ACE2, on the most representative antibodies. Among these sdAbs, results showed that the most potent antibody, n3130, could neutralize >90% SARS-CoV-2 pseudovirus at a concentration of 10 µg/ml, while n3088 could neutralize ~80% pseudovirus at a concentration of 10 µg/ml. No cytopathic effect (CPE) was observed in n3130 and only minimal signs of CPE were observed in n3088, while significant levels of CPE were detected in other antibody groups. However, none of these antibodies could effectively compete with ACE2 in binding the RBD of SARS-CoV-2 S protein.^49^

Based on the similarity of S protein between SARS-CoV-2 and SARS-CoV, researchers attempted to identify NMAbs with cross-reactivity for SARS-CoV-2 from the peripheral blood of SARS-infected patients. In order to detect the cross-reactivity of NMAbs against SARS-CoV-2, pseudovirus neutralization assays with a murine leukemia virus (MLV) pseudotyping system were performed. Results showed that S309 could effectively neutralize both pseudoviruses. By targeting the RBD (Fig. 6d), S309 potently neutralized authentic SARS-CoV-2 (2019n-CoV/USA_WA1/2020) with an IC_50_ of 79 ng/ml, via recognition of a highly conserved epitope in the S domain, comprising the N343-glycan.^28^

### RBD-specific cross-neutralizing MAbs against SARS-CoV and SARS-CoV-2

As discussed previously, some NMAbs can cross-bind the RBD of SARS-CoV and SARS-CoV-2, and some have cross-neutralization activity. The S protein sequence and crystal structure of SARS-CoV were found to be highly similar to those of SARS-CoV-2.^50–52^ Therefore, an affinity test of SARS-CoV NMAbs CR3022 and others was performed against the RBD of SARS-CoV-2. The results showed that only CR3022 had high affinity for the RBD of SARS-CoV-2,^53^ but it did not block binding of ACE2 to SARS-CoV-2 RBD (Fig. 6e). However, the authors of this study note that multiple antibodies have been reported that offer in vivo protection, even without in vitro neutralization activity.^54^ On the contrary, recent research results by scientists have shown that CR3022 has neutralizing activity against SARS-CoV-2 with IC_50_ of ~0.114 μg/ml in vitro.^55^ NMAbs 47D11, 18F3, VHH-72 and S309 derived from SARS-CoV have cross-neutralization activity to SARS-CoV-2 because they target highly conserved sites between SARS-CoV and SARS-CoV-2.^25,27,28,35^ Recently, a series of cross-antibodies were screened out from memory B cell repertoire of a convalescent SARS donor.^41^

## Conclusion and prospects

Whether coronaviruses, such as SARS-CoV and MERS-CoV, will threaten human health in the future is unknown. With the third pandemic of coronavirus pneumonia caused by SARS-CoV-2, this reminds us that the answer may be in the affirmative. Therefore, we need to develop prophylactic and therapeutic drugs against coronavirus infection as soon as possible. Antibody drugs with the characteristics of precise targeting, few side effects and significant curative effect are good choices for the treatment of coronavirus infection, and the RBD is the most important therapeutic target.

In this article, we have summarized NMAbs that target the RBD of SARS-CoV and SARS-CoV-2 and may have prophylactic or therapeutic potency against SARS and COVID-19. The sources, antiviral activity and targets of NMAbs are compared in Tables 2 and 3. A body of evidence suggests that antibodies targeting conserved sites have more broad-spectrum antiviral activity. Therefore, antibodies that target conserved sites are more likely to become broad-spectrum antibodies for coronaviruses that have already appeared or will appear in the future. From these studies, we understand that the antiviral ability of a single NMAb is limited, but that the combination of multiple NMAbs (cocktails) that target different epitopes can achieve satisfactory results in a synergistic way, limiting the possibility of virus escape. The recently reported NMAb 4A8, isolated from convalescent patients and targets N-terminal domain of SARS-CoV-2 S protein, has neutralizing activity against pseudoviruses with EC_50_ of 49 μg/ml and live viruses with EC_50_ of 0.61 μg/ml respectively,^56^ and could be used as a candidate of a cocktail. Indeed, therapeutic antibody cocktails have been used successfully for the treatment of patients infected with Ebola virus.^57^ Among NMAbs we discussed, there are some NMAbs whose sequences are remarkable closed to germline antibodies (e.g., ab1, 2–15),^32,48^ without extensive somatic hypermutations. This bodes well for vaccine development, and these NMAbs have more potency for therapy.

**Table 2:** Neutralizing monoclonal antibodies against SARS-CoV

NMAb’s name	Type	Source	Preparation	Target	Assay and result	Ref.
80R	sdAb	Human	Nonimmune phage libraries of human antibodies	324–503	LV neutralization: IC_50_ < 7.43 nM;	^13,14^
					Full protection of mice: 4/4 mice with >4 log reduction in lung viral load at 12.5 mg/kg.	
CR3014	scFv	Human	Semisynthetic antibody phage display libraries	318–510	LV neutralization: ND_100_ at 42 nM;	^15,16^
					Protection of ferrets: 10 mg/kg.	
1A5 2C5	IgG	Mouse	Animal immunization; hybridoma technology	310–535	LV neutralization: IC_50_ = 0.71 µg/ml.	^17^
					LV neutralization: IC_50_ = 1.27 µg/ml.	
S230.15	IgG	Human	B cell-based EBV transformation	RBD	Full protection of mice: 200 μg/mouse.	^18^
m396	IgG1	Human	Healthy human antibody libraries of volunteers	482–491	LV (GD03) neutralization: IC_50_ = 0.1 µg/ml;	^18^
					LV (Tor2) neutralization: IC_50_ = 0.01 µg/ml;	
					Full protection of mice: 200 μg/mouse.	
201	IgG1	Human	Transgenic mice; hybridoma technology	490–510	LV neutralization: IC_50_ ∼ 1 nM;	^20^
					Full protection of mice: 40 mg/kg.	
chimeric F26G18	IgG	Chimeric antibody	Animal immunization; hybridoma technology	460–476	LV neutralization: titer = 2.07 nM.	^22,23^
341 C	IgG	Mouse	Animal immunization; hybridoma technology	490–510	LV neutralization: ND_100_ at a dilution of 1:1280.	^24^
VHH-72	sdAb	llama	Animal immunization and sequencing	RBD	PsV neutralization: IC_50_ = 0.14 µg/ml.	^25^
CR3022	scFv	Human	Semisynthetic antibody phage display libraries	RBD	LV neutralization: IC_100_ = 23.5 µg/ml.	^26^
47D11	IgG	Chimeric antibody	Transgenic mice; hybridoma technology	RBD	PsV neutralization: IC_50_ = 0.061 μg/ml;	^27^
					LV neutralization: IC_50_ = 0.19 μg/ml.	
S309	IgG	human	Peripheral blood of SARS-infected patients	the front end of RBD	PsV neutralization: IC_50_ = 120 ~ 180 ng/ml.	^28^
ADI-55689	IgG	Human	Memory B cell repertoire of a convalescent SARS donor	RBD	PsV neutralization: IC_50_ = 0.004 ~ 0.06 μg/ml.	^41^
ADI-55993						
ADI-56000						
ADI-55688						
ADI-56046						
ADI-56010						
ADI-55690						
ADI-55951						

*PsV* pseudovirus, *LV* live virus

**Table 3:** Neutralizing monoclonal antibodies against SARS-CoV-2

NMAbs’s name	Type	Source	Preparation	Target	Assay and result	Ref.
3F11	sdAb	Human	Humanized phage display library	RBD	PsV neutralization: IC_50_ = 3.8 ng/ml;	^29^
					LV neutralization: IC_50_ = 436 ng/ml.	
BD-368-2	IgG	Human	B cells of convalescent patients; Single cell sequencing	RBD	PsV neutralization: IC_50_ = 1.2 ng/ml;	^31^
					LV neutralization: IC_50_ = 15 ng/ml;	
					Full protection of mice: 20 mg/kg.	
ab1	IgG	Human	Phage displayed Fab, scFv and VH libraries	RBD	Reporter Gene neutralization assay: 200 ng/ml;	^32^
					LV neutralization: ND_100_ < 400 ng/ml;	
					Full protection of mice: 0.3 mg of IgG1 ab1.	
CB6	IgG	Human	B cells of convalescent patients	RBD	PsV neutralization: ND_50_ = 0.036 μg/ml;	^33^
					LV neutralization: ND_50_ = 0.036 μg/ml;	
					Protection of rhesus macaques: 50 mg/kg.	
B38	IgG	Human	Peripheral blood of SARS-CoV-2- infected patients	RBD	LV neutralization: IC_50_ = 0.177 µg/ml;	^34^
					Protection of mice: Lung viral load reduced by 32.8% compared with PBS control.	
H4	IgG	Human	Peripheral blood of SARS-CoV-2- infected patients	RBD	LV neutralization: IC_50_ = 0.896 µg/ml;	^34^
					Protection of mice: Lung viral load reduced by 26% compared with PBS control.	
7B11 18F3	IgG	Mouse	Animal immunization; hybridoma technology	RBD	PsV neutralization: IC_80_ = 10 μg/ml.	^35^
					PsV neutralization: IC_80_ = 10 μg/ml.	
P2C-1F11	IgG	Human	Plasma of convalescing patients	RBD	PsV neutralization: IC_50_ = 0.03 µg/ml.	^36^
rRBD-15	IgG	Human	A synthetic human Fab antibody library	RBD	PsV neutralization: IC_50_ = 12.2 nM.	^37^
VHH-72-Fc	HCAb	llama	Animal immunization and sequencing	RBD	PsV neutralization: IC_50_ ~ 0.2 µg/ml.	^25^
311mab–31B5 311mab–32D4	IgG	Human	B cells of convalescent patients	RBD	PsV neutralization: IC_50_ = 33.8 ng/ml.	^38^
					PsV neutralization: IC_50_ = 69.8 ng/ml.	
CC12.1	IgG	Human	B cells of convalescent patients	RBD	PsV neutralization: IC_50_ = 0.019 μg/ml;	^39^
					LV neutralization: IC_50_ = 0.022 μg/ml;	
					Full protection of Syrian hamsters: Antibody serum concentration of ~22 µg/ml.	
COVA1-18 COVA2-15	IgG	Human	B cells of convalescent patients	RBD	PsV neutralization: IC_50_ = 8 ng/ml;	^40^
					LV neutralization: IC_50_ = 7 ng/ml.	
					PsV neutralization: IC_50_ = 8 ng/ml;	
					LV neutralization: IC_50_ = 9 ng/ml.	
47D11	IgG	Chimeric antibody	Transgenic mice; hybridoma technology	RBD	PsV neutralization: IC_50_ = 0.061 μg/ml;	^27^
					LV neutralization: IC_50_ = 0.57 μg/ml.	
S309	IgG	human	Peripheral blood of SARS-infected patients	RBD	PsV neutralization: IC_50_ = 3.5 nM;	^28^
					LV neutralization: IC_50_ = 79 ng/ml.	
ADI-55689	IgG	Human	Memory B cell repertoire of a convalescent SARS donor	RBD	PsV neutralization: IC_50_ = 0.05 ~ 1.4 μg/ml;	^41^
ADI-55993					LV neutralization: showed neutralizing activity at 100 nM.	
ADI-56000						
ADI-55688						
ADI-56046						
ADI-56010						
ADI-55690						
ADI-55951						
REGN10989 REGN10987 REGN10933 REGN10934	IgG	Human	Transgenic mice; Peripheral blood of SARS-CoV-2-infected patients; Next Generation Sequencing	RBD	PsV neutralization: IC_50_ = 7.23 pM;	^42^
					LV neutralization: IC_50_ = 7.38 pM.	
					PsV neutralization: IC_50_ = 40.6 pM;	
					LV neutralization: IC_50_ = 42.1 pM.	
					PsV neutralization: IC_50_ = 42.8 pM;	
					LV neutralization: IC_50_ = 37.4 pM.	
					PsV neutralization: IC_50_ = 54.4 pM;	
					LV neutralization: IC_50_ = 28.3 pM.	
H11-H4-Fc H11-D4-Fc	HCAb	llama	Phage display library	RBD	LV neutralization: ND_50_ = 6 nM.	^44^
					LV neutralization: ND_50_ = 18 nM.	
H014	IgG	Chimeric antibody	Animal immunization and phage display	RBD	PsV neutralization: IC_50_ = 3 nM; LV neutralization: IC_50_ = 38 nM; Protection of mice: Lung viral load reduced by about 10 ~100 folds compared with PBS control.	^45^
COV2-2196 COV2-2130	IgG	Human	Peripheral blood of convalescent patients	RBD	PsV neutralization: IC_50_ = 0.7 ng/ml;	^47^
					LV neutralization: IC_50_ = 15 ng/ml.	
					PsV neutralization: IC_50_ = 1.6 ng/ml;	
					LV neutralization: IC_50_ = 107 ng/ml.	
2–15	IgG	Human	Peripheral blood of COVID-19 patients	RBD	PsV neutralization: IC_50_ = 0.7 ng/ml;	^48^
					LV neutralization: IC_50_ = 5 ng/ml;	
					Protection of hamsters: Viral RNA copy numbers and infectious virus titers in lung tissues were reduced by 4 logs or more compared with PBS control.	
CR3022	IgG	Human	Gene cloning; Protein expression	RBD	LV neutralization: IC_50_ = ~ 0.114 μg/ml.	^55^
4A8	IgG	Human	Peripheral blood of convalescent patients	NTD	PsV neutralization: EC_50_ = 49 μg/ml;	^56^
					LV neutralization: EC_50_ = 0.61 μg/ml.	

*PsV* pseudovirus, *LV* live virus

The clinical prospects of fully human antibodies are obvious. Early studies mainly used EBV^58,59^ to immortalize B cells or fuse B cells with suitable partners to produce hybridoma.^60^ With scientific technology updates, now methods mainly used to obtain human antibodies include (i) humanizing mouse mAbs,^61^ (ii) screening for mAbs from phage display libraries of human antibody fragments,^62^ and (iii) immunizing transgenic mice carrying human immunoglobulin gene, followed by hybridoma technology for production of mAbs.^63^ In addition, screening of antibodies from patients is the most straightforward and simple method to obtain human antibodies as described above. As COVID-19 epidemic continues, more and more NMAbs have been isolated from the lymphocytes of COVID-19 patients. Although the neutralizing ability of convalescent plasma from some patients was relatively low, some NMAbs isolated from these patients had strong neutralizing activity with IC_50_ as low as 2 ng/ml against live SARS-CoV-2 infection,^64^ suggesting that using a vaccine, once it is available, to elicit NMAbs could be an alternative approach. Although numerous antibodies blockbuster antibody drugs are already on the market, technological innovation to improve antibody production is still needed, given that huge amount of anti-SARS-CoV-2 antibodies are required for the clinical application. Interestingly, direct antibody synthesis in combination with computerized and bioinformatic approaches are being developed, substantially shortening the time required for antibody development.^65–67^ At the same time, we look forward to new progress in the research and development of antibody drugs, the improvement in long half-life, high efficiency and miniaturization of antibody drugs, and the more economical development of antibody drugs.
